# Outcomes and Post-removal Course of Lumen-Apposing Metal Stent Placement for Peripancreatic Fluid Collections: A Comparative Study of Pancreatic Pseudocysts and Walled-Off Necrosis

**DOI:** 10.7759/cureus.71561

**Published:** 2024-10-15

**Authors:** Koji Takahashi, Hiroshi Ohyama, Izumi Ohno, Naoya Kato

**Affiliations:** 1 Gastroenterology, Chiba University, Chiba, JPN

**Keywords:** endoscopic ultrasound (eus), lumen-apposing metal stent, pancreatic fluid collection, pancreatic pseudocyst (ppc), walled-off necrosis

## Abstract

Aim: Pancreatic fluid collections (PFCs) are common local complications of pancreatitis that may require interventional therapy. Endoscopic ultrasound (EUS)-guided transluminal drainage from the digestive tract, particularly with lumen-apposing metal stents (LAMS), is the first-line therapy due to its safety and efficacy. However, adverse events and post-removal courses remain uncertain. This study aimed to clarify the characteristics of LAMS placement and its removal, comparing pancreatic pseudocysts (PPC) and walled-off necrosis (WON).

Methods: This single-center retrospective study included 23 patients who underwent transgastric LAMS placement for PFCs under EUS guidance. The patients were categorized into the PPC group (n = 14) and the WON group (n = 9). Backgrounds and clinical outcomes were analyzed and compared.

Results: The mean procedure time was 19 minutes in the PPC group and 25 minutes in the WON group, with no significant difference (p = 0.11). The overall incidence of adverse events during LAMS placement was 14.3% in the PPC group and 33.3% in the WON group, with no significant difference (p = 0.28), but the incidence of infection of noninfected fluid collections was 0% in the PPC group and 55.5% in the WON group, significantly higher in the WON group (p = 0.0016). At the time of LAMS removal, a double-pigtail plastic stent (DPS) was replaced in 53.8% of the PPC group and 57.1% of the WON group. Within one year, 57.1% of the replaced DPS in the PPC group and 25.0% in the WON group became dislocated. There were no adverse events due to the dislocation of the replaced DPS. Adverse events occurred in one patient in each group after LAMS removal: 7.7% in the PPC group and 14.3% in the WON group (p = 0.64), which consisted of peritonitis in the PPC group and recurrent infection of fluid collection in the WON group, and both of these events occurred when the replaced DPS was still in place after LAMS removal.

Conclusions: Although there was no significant difference in the overall incidence of adverse events between PPC and WON, the incidence of infection after LAMS placement was significantly higher in WON. Regarding the replaced DPS, there were some cases of dislocation within one year, but there were no related adverse events. Adverse events occurred even after the removal of LAMS and replacement with DPS, so careful follow-up is required.

## Introduction

Pancreatic fluid collections (PFCs) are common local complications of pancreatitis that may require interventional therapy [1]. Suitable candidates for drainage include symptomatic PFCs present for over four weeks, with well-formed walls, and located within 1 cm of the stomach or duodenum [2]. After four weeks from the onset of pancreatitis, PFCs without tissue necrosis are called pancreatic pseudocysts (PPC), and those with necrosis are called walled-off necrosis (WON). Endoscopic ultrasound (EUS)-guided transluminal drainage from the digestive tract is now considered the first-line therapy due to its safety, efficacy, and applicability compared to traditional methods, including percutaneous or surgical approaches [2,3]. In the transluminal approach, stents are placed to facilitate fluid drainage, followed by the creation of an anastomosis between the digestive tract and the PFC [2]. Lumen-apposing metal stents (LAMS) have emerged as an effective tool for EUS-guided drainage of PFCs. LAMS offer advantages such as a reduced risk of leaking fluid contents, faster drainage, and the ability to perform endoscopic debridement of infected necrotizing tissue [4]. The procedure to remove necrotizing tissue is called necrosectomy and is performed by inserting an endoscope through the LAMS into the lumen of the PFCs. However, adverse events associated with LAMS include bleeding, perforation, migration, and stent occlusion [5]. The characteristic saddle-shaped design of the LAMS allows for close contact between the gastrointestinal wall and the wall of the PFCs, and the large diameter lumen of the LAMS facilitates effective drainage [6]. As LAMS usage expands, endoscopists must be aware of potential adverse events and their management to ensure safe and successful outcomes [5]. LAMS is useful in treating symptomatic PFCs; however, many uncertainties remain regarding the associated adverse events and post-removal outcomes. In this study, we examined the course of LAMS placement in PFCs, including its removal and subsequent double-pigtail plastic stents (DPSs) replacement, dividing cases into PPC and WON, with the aim of clarifying their characteristics.

## Materials and methods

Study design

This single-center retrospective study included patients who underwent transgastric LAMS placement for PFCs under EUS guidance four weeks from the onset of pancreatitis at Chiba University Hospital between April 2019 and November 2023. A total of 23 cases were judged eligible, excluding those where transluminal drainage was performed prior to LAMS placement, cases with two or more transgastric drainage routes were created, and cases involving percutaneous biliary drainage. The remaining cases, in which treatment was performed only via a single transgastric LAMS route, were divided into the PPC group and the WON group. Baseline characteristics and clinical outcomes were compared. Baseline characteristics, clinical outcomes of LAMS placement, and clinical outcomes after LAMS placement were reviewed and compared. Background characteristics included age, sex, presence or absence of infection, cause of pancreatitis, maximum diameter of fluid collection, and amylase value of fluid collection. Clinical outcomes of LAMS placement included procedure time, details of plastic tube placed in the lumen of LAMS, adverse events after LAMS placement, percentage of necrosectomy performed during LAMS placement, and number of necrosectomies performed during LAMS placement. Clinical outcomes after LAMS placement included the number of cases in which LAMS removal was confirmed, the percentage of necrosectomy performed after LAMS removal, the duration of LAMS placement in cases of removal, the number of DPSs replaced at the time of LAMS removal, the method of removal of DPS replaced after LAMS, adverse events after LAMS removal, and the percentage of relapse of fluid collection after DPS removal.

Techniques

**Figure 1:**
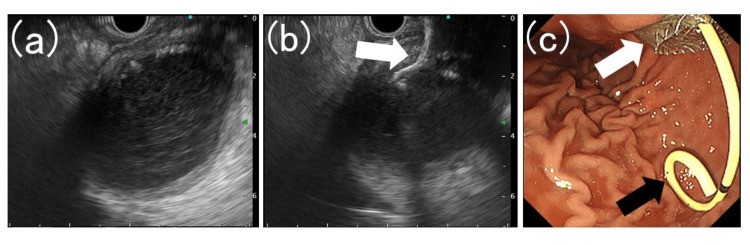
Placement of a lumen-apposing metal stent in the peripancreatic fluid collection and a double-pigtail stent in the lumen (a) Pancreatic fluid collection is observed in the stomach using an ultrasound endoscope. (b) Lumen-apposing metal stent (white arrow) is deployed by puncturing the peripancreatic fluid collection from the stomach under endoscopic ultrasound guidance. (c) A double-pigtail plastic stent (black arrow) was placed in the lumen of the lumen-apposing metal stent (white arrow).

**Figure 2:**
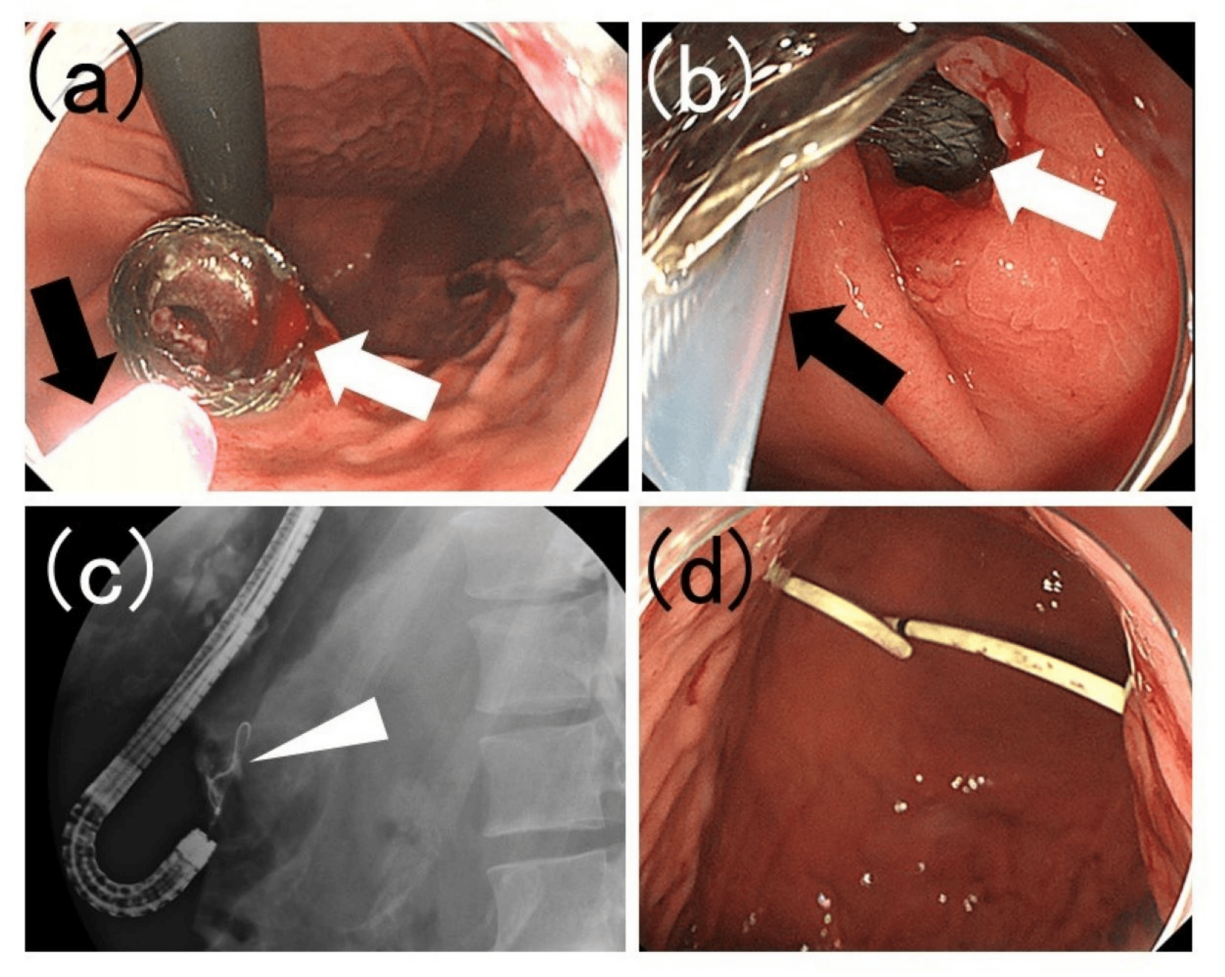
Removal of the lumen-apposing metal stent and replacement with a double-pigtail stent (a) The endoscope is brought closer to the lumen-apposing metal stent (white arrow), and the sheath of snare forceps (black arrow) is extending from the endoscope. (b) The lumen-apposing metal stent (white arrow) is grasped with a snare forceps (black arrow). (c) A guidewire (white arrowhead) is inserted into the cavity where the lumen-apposing metal stent was removed. (d) A double-pigtail plastic stent was placed using a guidewire in the cavity where the lumen-apposing metal stent had been placed.

All LAMS placements were performed transgastrically under EUS guidance with a GF-UCT260 (Olympus, Tokyo, Japan), a forward-oblique viewing endoscope. Before endoscopic procedures, prophylactic antibiotics were administered, and carbon dioxide was used for insufflation during endoscopic procedures. The decision of whether to place a DPS in the lumen of the LAMS when placing the LAMS and whether to replace the LAMS with a DPS when removing the LAMS was left to the discretion of the endoscopist. A Hot AXIOS (Boston Scientific Corp., Natick, USA) with a 15-mm inner diameter was used as the LAMS device in all eligible cases. The DPS to be placed in the lumen of the LAMS at the same time as placement, or to be replaced when the LAM was removed, was Advanix J (Boston Scientific Corp., Natick, USA) or Through & Pass (GADELIUS MEDICAL, Tokyo, Japan). It is the policy of our institution to remove the LAMS within 60 days of placement and to remove the DPS replacement with the LAMS endoscopically within one year (Figures 1, 2).

Definitions

**Figure 3:**
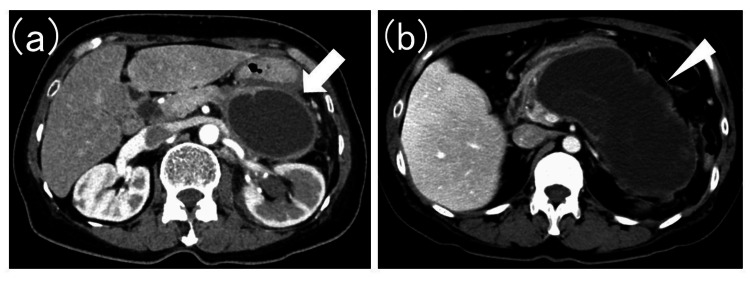
Computed tomography images of pancreatic pseudocyst and walled-off necrosis (a) Pancreatic pseudocyst (white arrow). (b) Walled-off necrosis (white arrowhead).

The distinction between PPC and WON was made comprehensively based on CT images, taking into account morphology and the presence or absence of necrotic tissue (Figure 3).

The maximum diameter of the fluid collection was measured using computed tomography images. Amylase levels in the fluid collection were measured from specimens obtained by inserting a catheter into the lumen of the LAMS after placement and aspirating the fluid. Procedure time was recorded from the insertion of the endoscope through the mouth until its removal. Adverse events related to endoscopic procedures were classified according to the severity grading system of the American Society for Gastrointestinal Endoscopy lexicon [7]. Relapse of fluid collection was defined as the re-enlargement of a previously reduced fluid collection, accompanied by the recurrence of symptoms, making it necessary to perform drainage again.

Statistical analysis

Data are presented as the mean with standard deviation or as numbers with percentages. Continuous variables were compared using the Mann-Whitney U test, while categorical variables were analyzed using Pearson's chi-squared test. A p-value of <0.05 was considered statistically significant. All analyses were conducted using BellCurve for Excel (Social Survey Research Information Co., Ltd., Tokyo, Japan).

Ethical statement

This study was approved by the Institutional Review Board of Chiba University Hospital (protocol code: HK202403-12 and date of approval: April 17, 2024) and conducted in accordance with the latest revision of the Declaration of Helsinki. Written informed consent for the procedure was obtained from all patients, and consent for study participation was secured through an opt-out methodology.

## Results

Of the 23 eligible cases, 14 were classified as PPC and nine as WON. Their background characteristics are shown in Table 1.

**Table 1:** Background characteristics of the two groups PPC: pancreatic pseudocyst; WON: walled-off necrosis; SD: standard deviation Statistical significance was defined as p < 0.05 by the Mann–Whitney U test.

	PPC (n = 14)	WON (n = 9)	p-value
Age, years, mean (SD)	57 (16)	56 (13)	0.90
Male, n (%)	7 (50.0%)	7 (77.8%)	0.18
Presence of infection, n (%)	2 (14.3%)	3 (33.3%)	0.28
Cause of pancreatitis, n (%)			
Alcohol	8 (57.1%)	3 (33.3%)	0.26
Tumor	3 (21.4%)	1 (11.1%)	0.52
Gallstones	1 (7.1%)	1 (11.1%)	0.74
Idiopathic	0	3 (33.3%)	0.021
Lipid dysfunction	0	1 (11.1%)	0.20
Autoimmune	1 (7.1%)	0	0.41
Retroperitonitis	1 (7.1%)	0	0.41
Maximum diameter of fluid collection, mm, mean (SD)	100 (34)	138 (46)	0.044
Amylase value of fluid collection, mg/dL, mean (SD)	54861 (67260)	28311 (39253)	0.051

There were no significant differences between the two groups in age, sex, or proportion of PFC infections before LAMS placement. There were no significant differences between the two groups in the causes of pancreatitis, except for idiopathic pancreatitis, which was significantly more common in the WON group. The maximum diameter of the fluid collection was significantly larger in the WON group. Amylase levels during fluid collection tended to be higher in the PPC group, but the difference was not significant.

The clinical outcomes during LAMS placement are summarized in Table 2.

**Table 2:** Clinical outcomes of lumen-apposing metal stent placement in the two groups PPC: pancreatic pseudocyst; WON: walled-off necrosis; SD: standard deviation; LAMS: lumen-apposing metal stent; DPS: double-pigtail plastic stent Statistical significance was defined as p < 0.05 by the Mann–Whitney U test.

	PPC (n = 14)	WON (n = 9)	p-value
Procedure time, minutes, mean (SD)	19 (9)	25 (9)	0.11
Plastic tube placed in the lumen of LAMS, n (%)			
None	6 (42.9%)	3 (33.3%)	0.65
DPS only	6 (42.9%)	3 (33.3%)	0.65
Nasal drainage tube only	1 (7.1%)	2 (22.2%)	0.29
Both nasal drainage tube and DPS	1 (7.1%)	1 (11.1%)	0.74
Adverse events, n (%)	2 (14.3%)	3 (33.3%）	0.28
Peritonitis	2 (14.3%)	0	0.24
Infection excitation into non-infectious fluid collection	0	3 (33.3%)	0.021
Necrosectomy performed during LAMS placement, n (%)	0	5 (55.5%)	0.0016
Number of necrosectomies performed during LAMS placement, n (%)			
1	0	1 (11.1%)	0.20
2	0	1 (11.1%)	0.20
3	0	2 (22.2%)	0.065
10	0	1 (11.1%)	0.20

There were no significant differences in procedure time or the contents of the tube placed in the cavity during LAMS placement. There was no significant difference in the overall incidence of adverse events during LAMS placement, but infection excitation in noninfected fluid collections was significantly more frequent in the WON group. Necrosectomy during LAMS placement was not performed in any cases in the PPC group but was only performed in the WON group.

Table 3 shows clinical outcomes after the LAMS placement of the two groups.

**Table 3:** Clinical outcomes after lumen-apposing metal stent placement of the two groups PPC: pancreatic pseudocyst; WON: walled-off necrosis; LAMS: lumen-apposing metal stent; SD: standard deviation; DPS: double-pigtail plastic stent; -: Chi-squared test result not calculable Statistical significance was defined as p < 0.05 by the Mann–Whitney U test.

	PPC (n = 14)	WON (n = 9)	p-value
Cases in which LAMS removal was confirmed, n (%)	13 (92.9%)	7 (77.8%)	0.29
Necrosectomy performed after LAMS removal, n (%)	0	1 (11.1%)	0.20
Duration of LAMS placement in cases of removal, days, mean (SD)	54 (21)	53 (14)	0.66
Number of DPSs replaced at the time of LAMS removal, n (%)			
0	6 (46.2%)	3 (42.9%)	0.89
1	3 (23.1%)	3 (42.9%)	0.36
2	4 (30.8%)	1 (14.3%)	0.42
Method of removal of replaced DPS after LAMS, n (%)			
Dislocation	4 (57.1%)	1 (25.0%)	0.30
Removed with endoscope	2 (28.6%)	3 (75.0%)	0.14
Died with stent in place	1 (14.3%)	0	0.43
Adverse events after LAMS removal, n (%)	1 (7.7%)	1 (14.3%)	0.64
Recurrence of infection in a fluid collection	0	1 (14.3%)	0.16
Peritonitis	1 (7.7%)	0	0.45
Relapse of fluid collection after DPS removal, n (%)	0	0	-

There was no significant difference in the proportion of cases in which LAMS removal was confirmed between the two groups. Necrosectomy after LAMS removal was performed in only one case (11.1%) in the WON group. There were no significant differences in the number of days LAMS was in place, the number of DPS replaced at the time of LAMS removal, the removal method of the replaced DPS replaced from the LAMS, or the incidence of adverse events after LAMS removal. Regarding the replaced DPS, 57.1% in the PPC group and 25.0% in the WON group were dislocated within one year. There were no adverse events due to the dislocation of the replaced DPS. Adverse events after LAMS removal included peritonitis in one case (7.7%) in the PPC group and recurrence of infection in a fluid collection in one case (14.3%) in the WON group.

## Discussion

In this study, we examined the clinical course of PFCs following LAMS placement and subsequent DPS replacement, dividing the cases into PPC and WON to clarify their characteristics. Our study revealed the following: although there was no significant difference in the overall incidence of adverse events between PPC and WON, the incidence of infection after LAMS placement was significantly higher in cases of WON. In addition, adverse events occurred even after the removal of LAMS.

In the initial EUS-guided transluminal drainage of PFCs, there are two types of stents: LAMS and DPS. Several meta-analyses have been published comparing LAMS and DPSs in the initial EUS-guided transluminal drainage of PFCs. In 2021, Calo et al. reported that LAMS had superior clinical efficacy compared to DPS for WON, with a comparable incidence of adverse events [8]. Also in 2021, Lyu et al. reported that LAMS had higher clinical efficacy than DPS for PFCs but may lead to perforation in WON [9]. In 2022, Guzman-Calderon et al. reported that LAMS and DPS had similar clinical efficacy, though LAMS had a lower incidence of adverse events [10]. In 2023, Khizar et al. reported that LAMS had superior clinical efficacy and fewer adverse events compared to DPS for WON [11]. Finally, in 2024, Bang et al. reported that LAMS resulted in shorter procedure times than DPS for WON, but other clinical outcomes were unchanged [12]. These studies reflect a mixed consensus, with some showing LAMS superiority and others suggesting equivalent outcomes between LAMS and DPS. Furthermore, two meta-analyses reached opposing conclusions about the necessity of simultaneously placing DPSs in the lumen of LAMS when placing a LAMS. In 2023, Beran et al. reported that placement of DPSs in the lumen of LAMS does not improve clinical outcomes or reduce adverse events [13]. However, in 2024, Gopakumar et al. reported that placement of DPSs in the lumen of LAMS does reduce the rate of adverse events without affecting clinical outcomes [14]. The frequency of adverse events associated with LAMS placement varies among reports, but the most common include bleeding, obstruction, and migration [8-12].

In our study, the amylase value of fluid collection tended to be higher in PPC cases, though not significantly. In 2017, Watanabe et al. reported that higher amylase values of fluid collection were significantly associated with recurrence after EUS-guided drainage of PFCs [15]. Although the overall incidence of adverse events during LAMS placement did not significantly differ between groups, infection of non-infected fluid collections was significantly more frequent in the WON group. In terms of content, compared to PPC, which is mostly liquid, WON contains a lot of solid components, which make it difficult to excrete, making it more susceptible to infection, which is presumably why these results were obtained. In one case in which infection recurred after LAMS removal, we speculate that the cause of the recurrence of infection was insufficient necrosectomy. In cases of WON, removal of LAMS without performing sufficient necrosectomy is considered to be a risk for infection recurrence. However, since 44.4% of WON cases involved LAM removal without performing necrosectomy at all in our study, it is difficult to judge the extent to which necrosectomy should be performed.

At the time of LAMS removal, more than half of the patients in both groups had DPS replacement, and dislocation of the replaced DPS occurred often within one year, in our study. However, no adverse events related to prolapse were observed. While adverse events occurred in two cases after LAMS removal, in both cases, a replaced DPS was currently in place. It emphasizes the importance of being aware that adverse events can occur not only during and after LAMS placement but also after removal. When replacing a LAMS with a DPS, it is crucial to explain both the benefits and the risks to the patient. Although dislocation of the DPS occurred frequently, no adverse events related to this dislocation were observed.

This study has several limitations. First, the sample size was relatively small. Second, the study included only cases treated via a single transgastric LAMS route, which may limit the generalizability of the results.

## Conclusions

Although there was no significant difference in the overall incidence of adverse events between PPC and WON, the incidence of infection after LAMS placement was significantly higher in WON. Regarding the replaced DPS, there were some cases of dislocation within one year, but there were no related adverse events. Adverse events occurred even after the removal of LAMS and replacement with DPS, so careful follow-up is required.
